# Decline in the proportion of methicillin resistance among *Staphylococcus aureus* isolates from non-invasive samples and in outpatient settings, and changes in the co-resistance profiles: an analysis of data collected within the Antimicrobial Resistance Surveillance Network, Germany 2010 to 2015

**DOI:** 10.1186/s12879-017-2271-6

**Published:** 2017-02-23

**Authors:** Jan Walter, Ines Noll, Marcel Feig, Bettina Weiss, Hermann Claus, Guido Werner, Tim Eckmanns, Julia Hermes, Muna Abu Sin

**Affiliations:** 1 Robert Koch Institute, Department for Infectious Disease Epidemiology, Postfach 65 02 61, D-13302 Berlin, Germany; 2 National Reference Centre for Staphylococci and Enterococci; Unit 13 Nosocomial Pathogens and Antibiotic Resistances; Robert Koch Institute, Burgstr. 37, D-38855 Wernigerode, Germany

**Keywords:** Antimicrobial resistance, Methicillin-resistant *Staphylococcus aureus* (MRSA), Surveillance, Livestock-associated MRSA, Germany

## Abstract

**Background:**

Recent analysis of trends of non-invasive infections with methicillin resistant *Staphylococcus aureus* (MRSA), of trends of MRSA infections in outpatient settings and of co-resistance profiles of MRSA isolates are scarce or lacking in Germany.

**Methods:**

We analysed data from the Antimicrobial Resistance Surveillance Network (ARS). We included in the analysis the first isolate of *S. aureus* per patient and year, which had a valid test result for oxacillin resistance and which was not a screening sample. We limited the analysis to isolates from facilities, which contributed to ARS for all six years between 2010 and 2015. We compared the proportion of methicillin resistance among *S. aureus* isolates by calendar year using Chi-square and Fisher’s exact test. We corrected for multiple testing using the Bonferroni correction. We stratified the analysis by sample type including various non-invasive sample types and by type of care (e.g. hospital versus outpatient clinic). We also analysed the non-susceptibility of MRSA to selected antibiotics.

**Results:**

The analysis included 148,561 *S. aureus* isolates. The distribution of these isolates by sex, age, region, sample type, clinical speciality and type of care remained relatively stable over the six years analysed. The proportion of MRSA among *S. aureus* isolates decreased continuously from 16% in 2010 to 10% in 2015. This decrease was seen for all types of care and for the majority of sample types, including the outpatient clinic (12 to 8%), as well as blood culture (19 to 9%), urine samples (25 to 15%), swabs (14 to 9%), respiratory samples (22 to 11%) and lesions (15 to 10%). The non-susceptibility of MRSA isolates to tobramycin (47 to 32%), ciprofloxacin (95 to 89%), moxifloxacin (94 to 84%), clindamycin (80 to 71%) and erythromycin (81 to 72%) declined markedly, but it increased for tetracyclines (6 to 9%) and gentamicin (3 to 6%). Non-susceptibility of MRSA to linezolid, teicoplanin, tigecycline and vancomycin remained rare.

**Conclusion:**

This analysis indicates that the incidence of MRSA infections declined in a variety of settings in Germany between 2010 and 2015 and that the co-resistance profiles of MRSA isolates changed markedly.

## Background

The incidence of infections with methicillin resistant *Staphylococcus aureus* (MRSA) is thought to be on the decline in Germany. This is corroborated by data from the national mandatory reporting system [1], from a hospital surveillance system [2], from surveys [3] and from analysis of data submitted to the European Antimicrobial Resistance Surveillance Network [4]. However, these analyses rely on invasive samples or on samples from hospitals only. Little is known about trends in the incidence of non-invasive MRSA infections and about that of MRSA infections in outpatient settings [5, 6].

The MRSA epidemic in Germany and Europe is thought to be mainly health-care associated based on the age distribution of cases [1] and molecular analysis of the isolates [7]. However, there are regions with a high density of swine farming, where livestock-associated (LA) MRSA makes up a considerable proportion of MRSA infections seen in hospitals. For example, one study found 8% of all MRSA blood cultures to be associated with LA-MRSA [8]. Community-associated (CA) MRSA is regularly found throughout Germany, even though it remains relatively rare [9]. Trends for LA- and CA-MRSA are not well reflected by the above mentioned surveillance systems, since this would require a more thorough data collection, ideally including the molecular characterization of isolates.

The Antimicrobial Resistance Surveillance Network (ARS) collects routine data on antibiotic resistance testing from voluntarily participating laboratories [10]. In contrast to other surveillance systems, ARS includes data on antibiotic susceptibility testing of *S. aureus* isolates from a number of sample types and settings including non-invasive samples and outpatient settings. It also includes data on co-resistance to other antibiotics for a large number of MRSA isolates. To complement recent analysis of declining MRSA infections in invasive samples and in hospitals [1–4] and to extend previous analysis from ARS [5, 11], we investigated whether declining trends of MRSA may be seen for all clinical settings, including outpatient settings, and for all sample types, including non-invasive samples. We further investigated whether or not there are changes in MRSA’s co-resistance profiles.

## Methods

### Study design

Among all data submitted to ARS, we selected facilities who submitted data for the entire study period from 2010 to 2015. We excluded screening samples (i.e. those marked as screening samples, those collected from swabs from the nose/throat and anal swabs) as well as those from unspecified sample types. We selected the first isolate of *S. aureus* per patient and year that had a valid test result for oxacillin (or to a comparable antibiotic, such as cefoxitin). We compared the proportion of *S. aureus* isolates that are resistant to oxacillin by year and stratified the analysis by type of care or by sample type. Among oxacillin resistant isolates we compared the proportion that were non-susceptible to other selected antibiotics by year.

### Statistical analysis

We grouped sample types into 7 groups: In addition to blood culture and urine, we included swabs from lesions and abscesses in one group (“lesions”). We grouped together swabs from eyes, ears, tonsils/throat, tongue, urogenital tract, those collected during surgery, as well as other and non-specified swabs (“swabs”). We also grouped together biopsies from tissues, liquor, abscesses, ascites, joints, pleural cavity, other and non-specified punctures (“biopsies”). We grouped together bronchial lavage, bronchial secretions, sputum, tracheal secretion and other respiratory samples (“respiratory sample”). The remaining samples were dialysate, ejaculate, skin, hairs, nails, catheters, faeces and unspecified samples (“other samples”). For the analysis of co-resistance, we used non-susceptibility as the outcome, grouping together resistant and intermediate test results. Due to cross-resistance between tetracycline and doxycycline, we grouped these two antibiotics together as the group of tetracyclines. In the analysis stratified by federal states, we excluded samples from several states with data for only few patients.

For univariate analysis we used chi-square tests unless the expected cell count was below 5, in which case we used Fisher’s exact test. We corrected for multiple testing using Bonferroni correction (i.e. dividing the p-value of 0.05 by the number of conducted tests [i.e. 27 for temporal differences in the frequency of oxacillin resistance; 6 for factors associated with non-susceptibility to tetracyclines]).

## Results

### Characteristics of included isolates

The analysis included 148,561 isolates from 6 laboratories representing 1,855 different outpatient clinics and 105 hospitals. Even though statistically significant, changes over the years were generally small for the distribution of the samples by category of age, sex, federal state of the sending facility, sample type, type of care or clinical speciality (Table 1).

**Table 1:** Number and characteristics of *S. aureus* isolates included in the analysis, ARS, Germany 2010-2015

	2010 [n (%)]	2011 [n (%)]	2012 [n (%)]	2013 [n (%)]	2014 [n (%)]	2015 [n (%)]	*p*-value
N	24 667	25 125	25 995	25 544	25 028	22 202	
Age [years]							<0.001
0–9	2 170 (9)	2 163 (9)	2 210 (9)	1 969 (8)	1 673 (7)	1 524 (7)	
10–19	1 321 (5)	1 335 (5)	1 461 (6)	1 319 (5)	1 199 (5)	1 105 (5)	
20–29	1 440 (6)	1 551 (6)	1 522 (6)	1 531 (6)	1 501 (6)	1 333 (6)	
30–39	1 349 (5)	1 394 (6)	1 378 (5)	1 369 (5)	1 269 (5)	1 203 (5)	
40–49	2 049 (8)	2 175 (9)	2 144 (8)	2 043 (8)	1 864 (7)	1 687 (8)	
50–59	2 721 (11)	2 736 (11)	2 945 (11)	2 892 (11)	2 878 (11)	2 588 (12)	
60–69	3 446 (14)	3 497 (14)	3 493 (13)	3 611 (14)	3 569 (14)	3 150 (14)	
70–79	5 439 (22)	5 491 (22)	5 714 (22)	5 771 (23)	5 657 (23)	4 830 (22)	
80+	4 732 (19)	4 783 (19)	5 128 (20)	5 039 (20)	5 418 (22)	4 782 (22)	
Sex							<0.001
Women	8 764 (36)	9 159 (36)	9 728 (37)	10 640 (42)	10 144 (41)	9 204 (41)	
Men	10 151 (41)	10 412 (41)	11 215 (43)	11 995 (47)	11 646 (47)	10 242 (46)	
Not specified	5 752 (23)	5 554 (22)	5 052 (19)	2 909 (11)	3 238 (13)	2 756 (12)	
Federal state							<0.001
Baden-Württemberg	4 634 (19)	4 844 (19)	4 709 (18)	4 882 (19)	2 839 (11)	2 886 (13)	
Bavaria	609 (2)	565 (2)	635 (2)	688 (3)	714 (3)	784 (4)	
Berlin	1 678 (7)	1 885 (8)	1 889 (7)	2 029 (8)	2 108 (8)	2 218 (10)	
Hesse	848 (3)	832 (3)	1 045 (4)	1 125 (4)	1 203 (5)	1 283 (6)	
North Rhine-Westphalia	11 778 (48)	11 838 (48)	12 465 (48)	12 758 (50)	13 809 (56)	10 652 (49)	
Rhineland Palatinate	2 387 (10)	2 322 (9)	2 351 (9)	2 301 (9)	2 512 (10)	2 397 (11)	
Schleswig-Holstein	2 511 (10)	2 598 (10)	2 659 (10)	1 524 (6)	1 622 (7)	1 728 (8)	
Sample type							<0.001
Swabs	9 352 (38)	9 383 (37)	9 720 (37)	8 819 (35)	8 669 (35)	8 077 (36)	
Blood cultures	1 109 (4)	1 172 (5)	1 263 (5)	1 262 (5)	1 250 (5)	1 093 (5)	
Biopsies	469 (2)	505 (2)	528 (2)	560 (2)	459 (2)	410 (2)	
Respiratory samples	2 537 (10)	2 523 (10)	2 624 (10)	2 687 (11)	2 508 (10)	2 345 (11)	
Urine	2 494 (10)	2 508 (10)	2 569 (10)	2 456 (10)	2 508 (10)	1 973 (9)	
Lesions	8 118 (33)	8 427 (34)	8 701 (33)	9 098 (36)	9 109 (36)	7 795 (35)	
Other	588 (2)	607 (2)	590 (2)	662 (3)	525 (2)	509 (2)	
Type of care							0.016
Outpatient care	10 846 (44)	11 537 (46)	11 588 (45)	11 506 (46)	10 811 (44)	10 209 (46)	
ICU	1 798 (7)	1 857 (7)	2 002 (8)	1 875 (7)	1 716 (7)	1 620 (7)	
Normal hospital ward	11 920 (49)	11 600 (46)	12 104 (47)	11 793 (47)	12 001 (49)	10 223 (46)	
Clinical specialty							<0.001
Surgery and related	5 208 (21)	5 040 (20)	5 226 (20)	5 047 (20)	4 832 (19)	3 843 (17)	
Internal/conservative	12 160 (49)	12 339 (49)	12 652 (49)	12 620 (49)	12 355 (49)	10 949 (49)	
Other	7 299 (30)	7 746 (31)	8 117 (31)	7 877 (31)	7 841 (31)	7 410 (33)	

ICU = intensive care unit. The numbers may not tally to the total due to missing data or due to a low frequency of entries, which do not fit the categories shown (i.e. for type of care and for federal state)

### Oxacillin resistance

The overall frequency of oxacillin resistance decreased continuously from 16% (*n* = 4,058) in 2010, to 15% (*n* = 3,853) in 2011, 14% (*n* = 3,675) in 2012, 12% (*n* = 3,129) in 2013, 12% (*n* = 2,974) in 2014 to 10% (*n* = 2,223) in 2015 (*p* < =0.001).

**Fig. 1:**
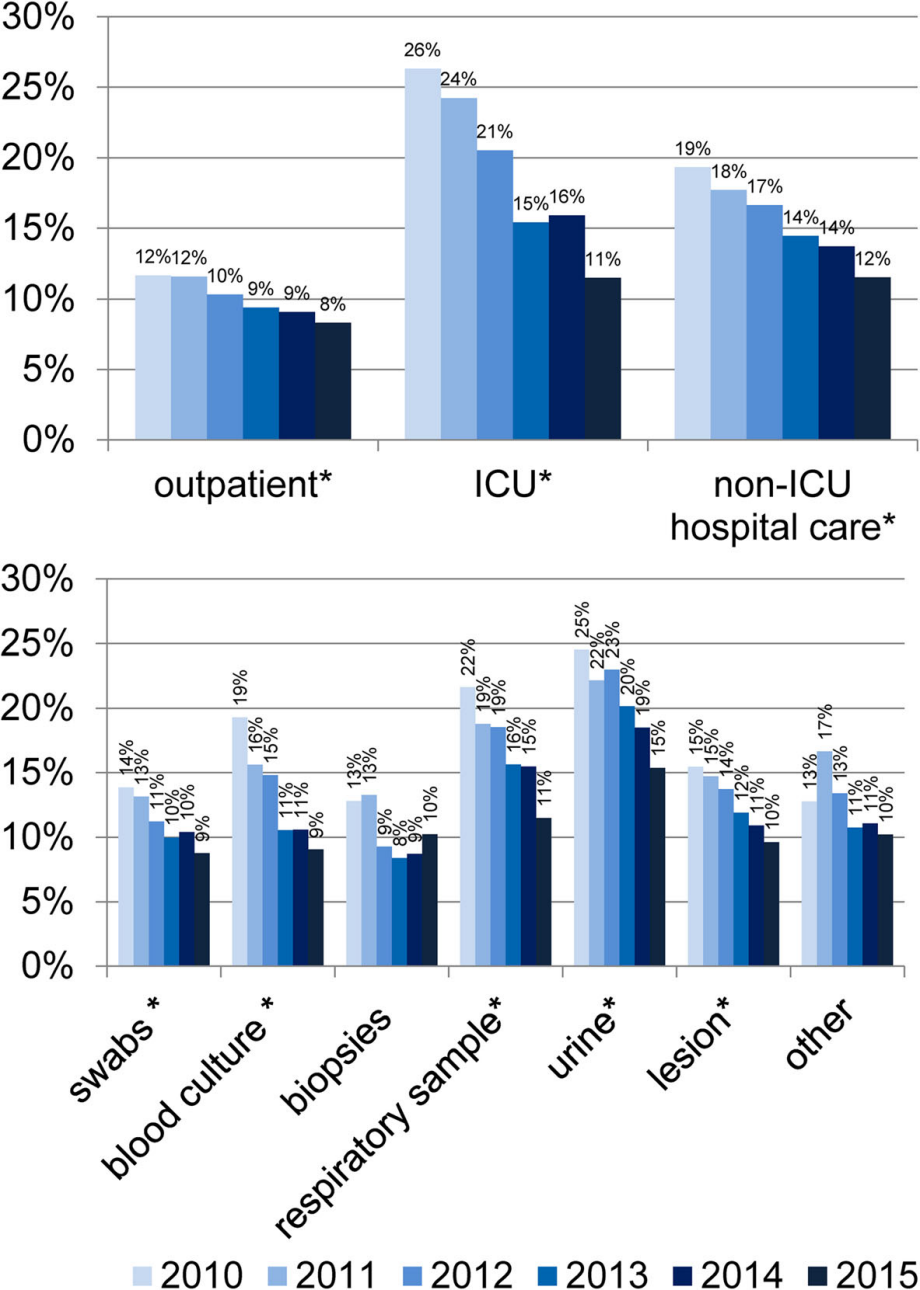
MRSA among *S. aureus* isolates by type of care or sample type, ARS, Germany, 2010–2015. Legend: The asterisk marks significant changes (*p* < 0.0019)

There were significant declines in the proportion of MRSA among all *S. aureus* isolates for all types of care (Fig. 1, upper panel). As expected, the proportion of MRSA was generally lower in the outpatient setting than in hospitals. However, the relative decline was markedly lower in the outpatient setting than in the hospital setting (12 to 8% between 2010 and 2015 versus 26 to 11% in ICU and 19 to 12% in non-ICU hospital wards), closing the gap between these two types of care.

There was a strong decline in the proportion of MRSA among *S. aureus* isolates for all sample types except for biopsies and other samples, for which the p-value did not reach statistical significance after adjustment for multiple testing (i.e. *p* < 0.0019) (Fig. 1, lower panel).

### Non-susceptibility of MRSA to other antibiotics

**Fig. 2:**
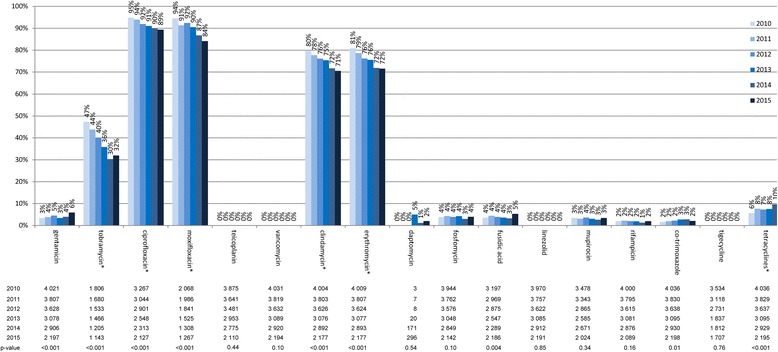
Non-susceptibility of MRSA isolates to selected antibiotics, ARS, Germany, 2010–2015. Legend: The table shows the number of tested isolates. The asterisk marks significant changes (*p* < 0.0019)

Figure 2 depicts the non-susceptibility of MRSA isolates to other selected antibiotics per calendar year. At least three different patterns can be identified: Firstly, antibiotics with high (>30%) but decreasing levels of non-susceptibility (i.e. tobramycin, ciprofloxacin, moxifloxacin, clindamycin, erythromycin); secondly, antibiotics with low (≤5%) and decreasing or stable levels of non-susceptibility (i.e. teicoplanin, vancomycin, daptomycin, fosfomycin, fusidic acid, linezolid, mupirocin, rifampicin, cotrimoxazole and tigecyline); thirdly, two antibiotics or groups of antibiotics with relatively low levels (<10%) but increasing levels of non-susceptibility (i.e. gentamicin and tetracyclines). The absolute numbers of isolates with non-susceptibility to gentamicin (2010 to 2015: *n* = 135, 145, 164, 104, 114 and 130) or to tetracyclines (2010 to 2015: *n* = 225, 293, 262, 233, 282 and 203) remained relatively stable over the years, due to the decline in the overall number of MRSA isolates.

Since non-susceptibility to tetracyclines has been found to be associated with LA-MRSA in several studies [12–18], we analysed the associated factors. We found non-susceptibility to tetracyclines to be associated with young age (Table 2). While there was no difference between the two sexes (*p* = 0.13), non-susceptibility was higher if for technical reasons the sex had not been specified. There were also significant differences between German federal states, however with no clear pattern. Non-susceptibility was relatively high among isolates from swabs, biopsies, lesions and other samples, but lower in isolates from blood culture. As expected it was higher in samples from outpatient settings than from hospitals (*p* = 0.004 for outpatient versus both hospital settings). Non-susceptibility to tetracyclines was more frequent among isolates from surgery than in those from other clinical specialities.

**Table 2:** Non-susceptibility of MRSA isolates to tetracyclines, Antimicrobial Resistance Surveillance Network – ARS, Germany, 2010–2015

	N MRSA isolates tested for tetracycline resistance	n (%) non-susceptible to tetracyclines	*p*-value
N	19 721	1 498 (8)	
Age [years]			<0.001
0–9	414	79 (19)	
10–19	216	45 (21)	
20–29	367	64 (17)	
30–39	436	75 (17)	
40–49	936	107 (11)	
50–59	1 883	180 (10)	
60–69	3 219	252 (8)	
70–79	6 093	357 (6)	
80+	6 157	339 (6)	
Sex			<0.001
Women	6 449	428 (7)	
Men	8 736	635 (7)	
Unspecified	4 536	435 (10)	
Federal state			<0.001
Baden-Württemberg	2 544	134 (5)	
Bavaria	336	47 (14)	
Berlin	1 248	84 (7)	
Hesse	664	40 (6)	
North Rhine-Westphalia	11 445	942 (8)	
Rhineland Palatinate	1 790	86 (5)	
Schleswig-Holstein	1 534	159 (10)	
Sample type			<0.001
Swabs (other than from lesions)	6 059	498 (8)	
Blood culture	926	43 (5)	
Biopsies	302	32 (11)	
Respiratory sample	2 538	183 (7)	
Urine	3 006	166 (6)	
Lesion	6 455	541 (8)	
Other	435	35 (8)	
Type of care			0.016
Outpatient care	6 668	553 (8)	
Intensive care unit	2 048	142 (7)	
Normal hospital ward	10 780	776 (7)	
Clinical specialty			0.001
Surgery	3 943	348 (9)	
Internal/conservative	10 645	748 (7)	
Other	5 133	402 (8)	

The numbers may not tally to the total due to missing data or due to a low frequency of entries, which do not fit the categories shown (i.e. for type of care and for federal state)

## Discussion

The proportion of MRSA among *S. aureus* isolates from non-invasive samples and from those in the outpatient setting decreased significantly between 2010 and 2015. Similarly, non-susceptibility of MRSA isolates to several other antibiotics decreased between 2010 and 2015, while that to gentamicin and to tetracyclines increased. Non-susceptibility to last line antibiotics, including tigecycline, linezolid, vancomycin and teicoplanin, fortunately remained rare.

Our data are also consistent with previous analyses that have indicated declines in MRSA in the hospital setting and for invasive infections [1–3], with the previous analysis of ARS data from outpatient setting [5] and indirectly also with data from the national reference laboratory that suggest low levels of LA- and CA-MRSA in Germany [6, 9]. Therefore, they indicate a general decline in MRSA-infection in Germany in all settings and for all sample types.

A decline in MRSA infections in Germany may have various reasons as discussed previously [1]. It is likely that control mechanisms implemented in Germany contribute to this success. However, additional factors, such as normally occurring changes in the circulating strains cannot be excluded [19].

Of note is the relative increase in the non-susceptibility of MRSA isolates to tetracyclines. Non-susceptibility to tetracyclines had been found to be associated with LA-MRSA [13–18]. We found it to be associated with –among other factors - young age and samples from outpatient settings, which would be consistent with LA-MRSA. Since, however, non-susceptibility to tetracyclines also occurs independently of LA-MRSA [12], we cannot with certainty attribute trends in non-susceptibility to tetracyclines to an increase in LA-MRSA. An increase in the proportion of LA-MRSA among all MRSA isolates would however be consistent with two previous studies that showed an increase of LA-MRSA between 2004/2005 and 2010/2011 [20] as well as stable levels of LA-MRSA in recent years [12] (while other forms of MRSA have declined). The relevance of LA-MRSA in Germany therefore warrants further investigations.

The proportion of MRSA among *S. aureus* isolates is an imperfect indicator for the incidence of MRSA infections, because it may be influenced by the frequency of diagnostic sampling [21], the time of sample collection, changes in treatment practice and because it does not include a defined population as denominator. As with all routine data from voluntary sources, an additional limitation of this analysis is the possibility of a changing study base and a potential non-representativeness of the data for all of Germany. Since, however, the baseline characteristics (Table 1) remained relatively stable over the time period included in the study and since the results for the blood cultures showed consistent trends with data from other sources [1, 2], we believe our results to be indicative for trends in the incidence of MRSA infections in Germany.

## Conclusion

The presented data from ARS corroborate a general decline of MRSA infections in Germany including in the outpatient setting and in non-invasive samples. The co-resistance profiles changed markedly and should be further analysed using genotyping studies.

## Abbreviations

ARS: Antimicrobial Resistance Surveillance Network
CA: Community-associated
LA: Livestock-associated
MRSA: Methicillin resistant *Staphylococcus aureus*
